# Identification of juvenility-associated genes in the mouse hepatocytes and cardiomyocytes

**DOI:** 10.1038/s41598-018-21445-3

**Published:** 2018-02-15

**Authors:** Faidruz Azura Jam, Yosuke Kadota, Anarmaa Mendsaikhan, Ikuo Tooyama, Masaki Mori

**Affiliations:** 1 Molecular Neuroscience Research Center (MNRC), Shiga University of Medical Science, Tsukinowa-cho, Seta, Otsu, Shiga, 520-2192 Japan; 2 Department of Systems BioMedicine, Tokyo Medical and Dental University, 1-5-45, Yushima, Bunkyo-ku, Tokyo, 113-8510 Japan

## Abstract

Young individuals possess distinct properties that adults do not. The juvenile animals show higher activities for growth, healing, learning and plasticity than adults. The machinery for establishing these juvenile properties is not fully understood. To better understand the molecular constituents for the above properties, we performed a comprehensive transcriptome analysis of differently aged cells of mice by high-throughput sequencing and identified the genes selectively highly expressed in the young cells. These genes, collectively called as juvenility-associated genes (JAGs), show significant enrichments in the functions such as alternative splicing, phosphorylation and extracellular matrix (ECM). This implies the juvenescence might be achieved by these functions at the cell level. The JAG mutations are associated with progeria syndromes and growth disorders. Thus, the JAGs might organize the juvenile property of young animals and analysis of JAGs may provide scientific and therapeutic approaches toward treating the genetic diseases.

## Introduction

Children are different from adults in numerous aspects. Young individuals can grow in size, maturate in function, learn faster and heal wounds more quickly. These properties are prominent in comparison to adults^1,2^. Their organs, including even the brain, exhibit maturational activity, functional plasticity and recovery after an injury^3–6^. These physiological properties, if utilized effectively, may contribute to an establishment of a new therapeutics for childhood-onset intractable diseases. The molecular building blocks underlying these juvenility-specific features, however, have not been systematically investigated.

Growth is one of the predominant characteristics of the juvenile properties. It is, however, not well understood how the growth is regulated and how organ growth stops at the particular set point^7–13^. In liver, hepatocytes cease cell division when the liver reaches the particular size, but resume proliferation once the liver size is decreased by, for example, a surgical resection and recuperate the original size^14–17^. Cardiomyocytes also show robust cell division in its infancy^18,19^, but the potential molecular machineries underlying the proliferative activities of the juvenile cardiomyocytes remain to be determined.

Maturation is another important characteristic of the postnatal physiology^20–22^. The liver exhibits functional refinement after birth so that it is capable of detoxifying toxins and oxidants, the stresses the juvenile cells face in the extracorporeal environment^23–27^. Genes contributing to the hepatic maturation are not comprehensively explored yet, though it accompanies with a rapid upregulation of mitochondrial enzymes such as succinic dehydrogenase and F1-ATPase^28^ and with activation of beta-catenin^29^.

The maturation in the heart accompanies with the robust proliferation of the differentiated cardiomyocytes^18,19^ and the cellular hypertrophy to meet the increasing cardiac load as a result of the body size increment^30–32^. A precise machinery for the cardiac maturation is not well understood either, besides the preceding paper clarified the switching of energy use from glycolysis to mitochondrial oxidation^33^.

The properties of the young organs have been previously assessed in gut^34,35^, lung^36^, skin^37^, and hemodynamics^38^. The comparison between juvenile and adult creatures were made in terms of human muscle physiology^39^, exercise tolerance^40^, mouse striatal neurons^41^, rat cerebral cortex^42^, dentate granule cells^43,44^ and eel digestive proteases^45^.

In this study, we aim at identifying the genes that constitute the physiological properties of the juvenile cells that has capacities for the growth and the maturation. For that purpose, we performed a transcriptome analysis to comprehensively identify the genes selectively expressed in the young cells. We selected 2 organs, the liver and the heart for the analysis based on two reasons: first, cell isolation techniques have been established to obtain the pure population of parenchymal cells from the organs. Second, these organs exhibit 3-dimensional (3D) growth that has not been molecularly defined well.

We here analyze a comprehensive transcriptome by high-throughput sequencing in the differently aged mouse hepatocytes and cardiomyocytes, to identify the specific gene sets as we call juvenility-associated genes (JAGs) constituting the juvenile properties of young organs. The JAGs show characteristic enrichments in the functions such as alternative splicing, phosphorylation and extracellular matrix (ECM), indicating these cellular functions are the important building blocks for the juvenile properties. The analysis of the JAGs provides a new approach for understanding how the juvenile properties are achieved and how the genetic diseases caused by the JAG mutation might be treated.

## Results

### Transcriptome analysis identifies the specific genes expressed in the juvenile hepatocytes and cardiomyocytes

**Figure 1:**
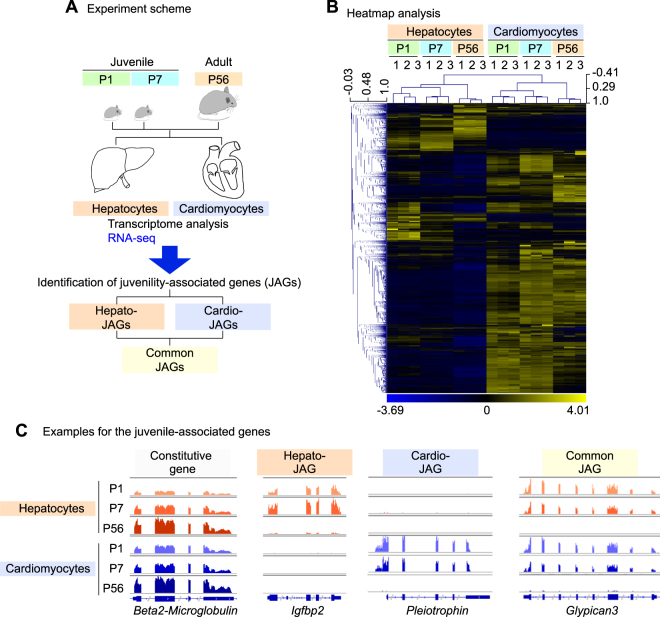
Transcriptome analysis reveals specific gene sets expressed in juvenile hepatocytes and cardiomyocytes in mice. (**A**) A schematic describing the identification of juvenility-associated genes (JAGs). Hepato-, hepatocyte. Cardio-, cardiomyocyte. P, postnatal day. (**B**) Heatmap analysis of the transcriptome analysis in the hepatocytes and the cardiomyocytes of mice at different ages. (**C**) Examples of a constitutively expressed gene (*Beta2-Microglobulin*), a hepato-JAG (*IGFBP2*), a cardio-JAG (*Pleiotrophin*) and a common JAG (*Glypican3*).

To identify the gene set expressed specifically highly in the young cells in the mouse organs, RNA-seq analysis was performed with the hepatocytes and cardiomyocytes isolated from mice at the postnatal days 1 and 7 (P1 and P7, the juvenile phase), and 56 (P56, the adult phase) (Fig. 1A and Supplementary Figure S1). The P1 is an early neonatal phase and the P7 corresponds to the late neonatal phase in mice. By observing at the 2 points (P1 and P7) as the juvenile phase, we aimed at delineating consistent alternations in comparison to the adult phase (P56) to capture the consistently relevant genes for the juvenile properties.

The heatmap analysis showed discrete gene expression signatures between the hepatocytes and the cardiomyocytes (Fig. 1B). This analysis revealed presence of the genes that were specifically expressed in the juvenile phase (Fig. 1C). For example, *IGFBP2* was expressed selectively in juvenile hepatocytes; *Pleiotrophin*, a growth factor, was expressed in juvenile cardiomyocytes; and *Glypican3* (*GPC3*) was expressed in both juvenile hepatocytes and cardiomyocytes (Fig. 1C). The RNA-seq results were validated with quantitative PCR analyses (Supplementary Figure S2). Thus, this transcriptome analysis identified the gene sets expressed predominantly in the juvenile cells. We collectively call these genes juvenility-associated genes (JAGs), aiming at identifying a specific function of the gene network that establishes the juvenile properties of the young cells.

### Identification of hepato-JAGs in mice

How the juvenile properties of the young hepatocytes are achieved is not well understood. The juvenile properties of the hepatocytes are seen in the robust cell proliferation and the distinct metabolic profiles^2,46–48^. The distinctiveness of the juvenile liver is also prominent in terms of the tumorigenicity. Hepatoblastoma is most common in juveniles, whereby the hepatocellular carcinoma and the biliary tumor, the common types in adults, are rare.

**Figure 2:**
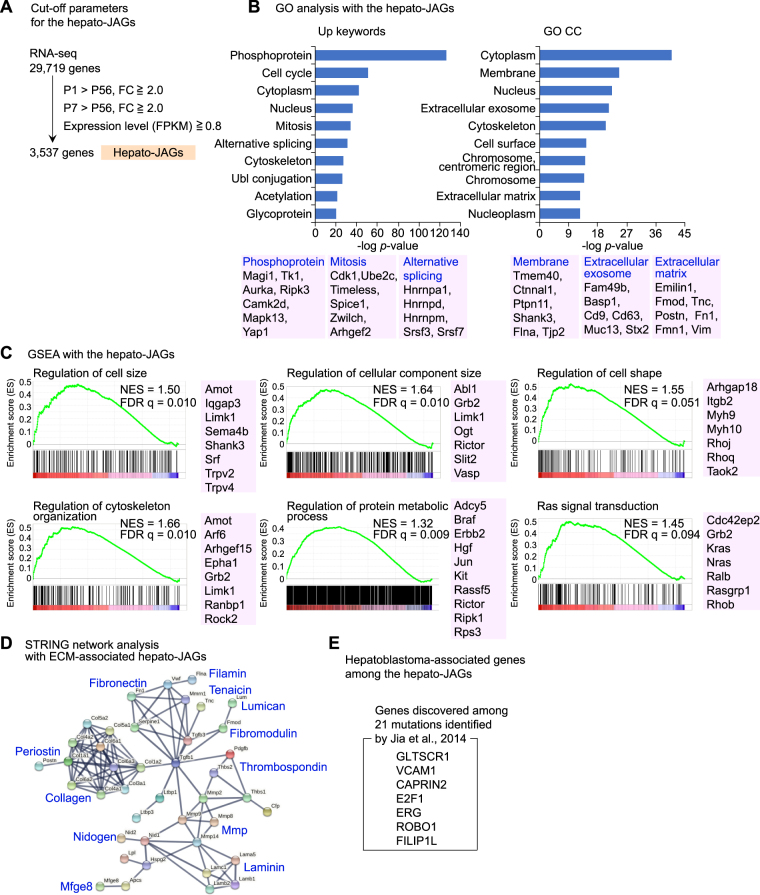
The hepato-JAGs exhibit a distinct gene expression profile. (**A**) Filtration and identification of hepato-JAGs from the comprehensive transcriptome analysis in the hepatocytes of the differently-aged mice. (**B**) The gene ontology (GO) analysis with the hepato-JAGs. GO CC, gene ontology cellular component. Ubl, ubiquitin and ubiquitin-like. (**C**) The gene set enrichment analysis (GSEA) with the transcriptome in the hepatocytes. NES, normalized enrichment score. FDR, false discovery rate. (**D**) The STRING network analysis with the extracellular matrix (ECM)-associated hepato-JAGs. (**E**) The hepato-JAGs discovered among the genes reported by Jia et al. to be causative for the hepatoblastoma.

To identify the genes that constitutes the molecular basis for the above features, we analyzed the hepatocyte transcriptome. Among all the 29,720 transcripts, 3,306 genes exhibited the expression levels higher than the cut off and the fold changes higher than 2.0 both at P1 and P7 comparing to P56 (Fig. 2A). To obtain an overview for the functions of the hepatocyte JAGs (hereafter hepato-JAGs), we performed a gene ontology (GO) analysis. The analysis showed the hepato-JAGs were associated significantly with the biological keywords such as phosphoprotein, cell cycle, mitosis and alternative splicing (Fig. 2B, Up keywords) and the cellular components (CC) such as cytoplasm, membrane, nucleus, extracellular exosome and extracellular matrix (ECM, Fig. 2B, GO CC). A gene set enrichment analysis (GSEA) showed significant enrichment of the hepato-JAGs into the functions such as cell and cellular component sizes, cell shape, protein metabolism and the Ras signaling (Fig. 2C). Numerous ECM genes were expressed highly in the juvenile hepatocytes, as depicted by the network generated by STRING interactome database (Fig. 2D). Thus, the transcriptome analysis identified hepatocyte JAGs that are the functional building blocks for the juvenile properties in the liver. To address if our analysis collects the physiologically relevant genes, we tested whether the hepato-JAGs possess a connection with pathogenesis in humans. A recent exome sequencing analysis with hepatoblastoma patients identified 21 genes that may causatively contribute to the tumorigenesis^49^. Among the genes, 7 genes (*GLTSCR1*, *VCAM1*, *CAPRIN2*, *E2F1*, *ERG*, *ROBO1* and *FILIP1L*) were included among the hepato-JAGs (Fig. 2E). This frequency is higher than the value predicted by chance (2.3 genes). This suggests a physiological relevance of the hepato-JAGs. Together, we identified the hepato-JAGs with distinctive expression and molecular functions in the juvenile hepatocytes.

### Maturation dynamics in the hepatocytes

**Figure 3:**
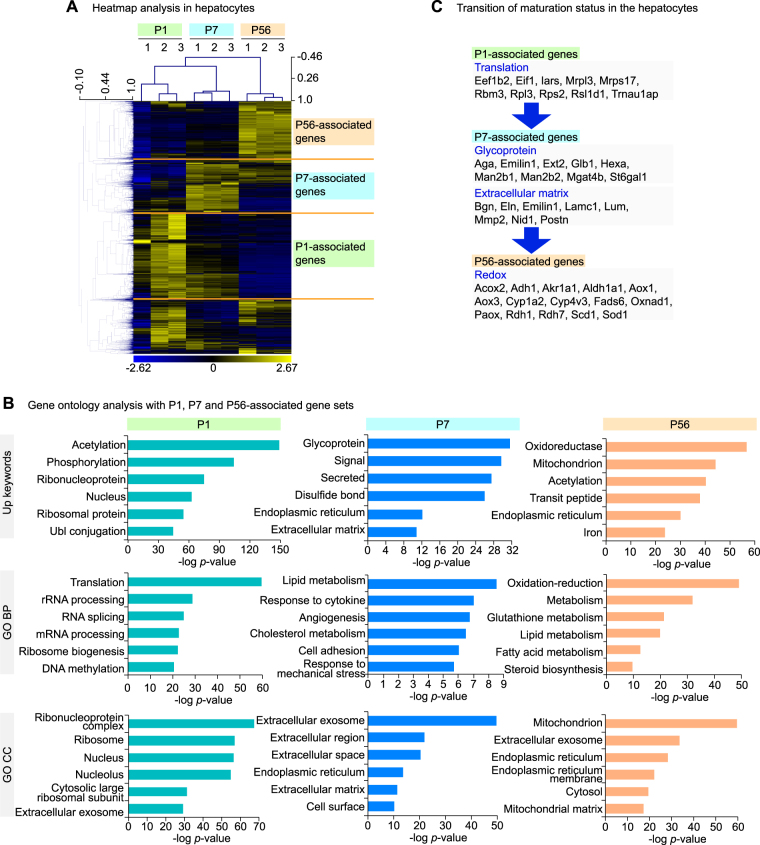
Maturation dynamics in the hepatocytes. (**A**) Heatmap analysis of transcriptome analysis in the hepatocytes at different ages. The distinct sets of genes were present as noted as P1-, P7- and P56-associated genes. (**B**) The GO analysis describing the transition of cellular functions in the hepatocytes. GO BP, gene ontology biological process. (**C**) A schematic for the transition of the cellular functions in the postnatal hepatocytes.

Maturation is a distinct feature of young animals, but its underlying molecular machinery is not well understood. To capture a mechanistic basis for the maturation, we delineated the genes expressed in each of P1, P7 and P56 timepoint, as P1-, P7- and P56-associated genes (Fig. 3A). The GO analysis with each gene set demonstrated that P1-associated genes were highly correlated with translation-related functions, such as “rRNA processing” and “ribonucleoprotein complex”. The P7-associated genes were linked with the glycosylation (“glycoprotein”) and extracellular matrix (“extracellular matrix”, “extracellular region”). P56-associated genes were linked with redox functions (“oxidoreductase”, “oxidation-reduction”, Fig. 3B). These findings revealed the transition of maturation status in the postnatal hepatocytes (Fig. 3C). Thus, the transcriptome analysis in the postnatal hepatocytes clarifies the maturation dynamics and the genes characterizing each maturation stage.

### Identification of cardio-JAGs in mice

**Figure 4:**
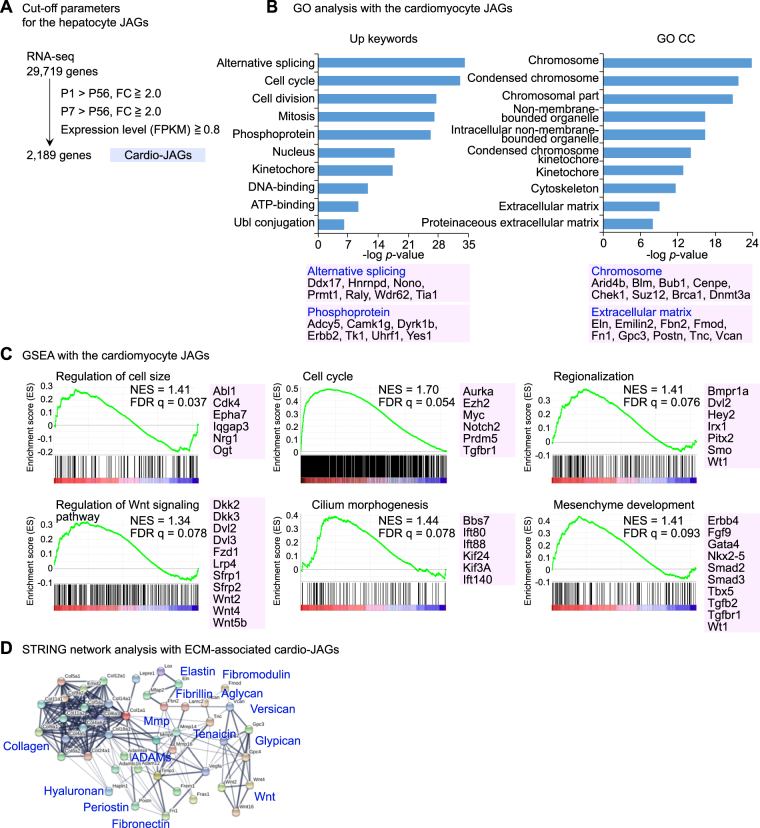
The cardio-JAGs exhibit a distinct gene expression profile. (**A**) Filtration and identification of cardio-JAGs from the comprehensive transcriptome analysis in the cardiomyocytes of the differently-aged mice. (**B**) The GO analysis with the cardio-JAGs. (**C**) The GSEA with the transcriptome in the cardiomyocytes. (**D**) The STRING network analysis with the extracellular matrix (ECM)-associated cardio-JAGs.

The juvenile properties of the cardiomyocytes are seen as their capacity for the cell divisions and the functional adaptability to the environmental necessities changing due to the birth and the increasing body size. The cardiomyocyte JAGs (hereafter cardio-JAGs) were identified from all the 29,720 transcripts based on the expression levels and fold changes comparing to adult cells (Fig. 4A). To gain an overall view as to their functions, we performed the GO analysis. The cardio-JAGs exhibit significant associations with keywords such as alternative splicing, cell cycle and phosphorylation and the cellular components such as chromosome and ECM (Fig. 4B). The inclusion of *UHRF1* and DNA methyltransferases (*Dnmt1/3a/3b*) implied a mechanism for the duplication of differentiated cardiomyocytes (detailed in Discussion). The highly significant association of the ECM facilitated us to list up the ECM-related genes in the cardio-JAGs as depicted by the STRING database-generated interactive network (Fig. 4C). Also, the GSEA showed significant association with functions such as “cell size”, “cell cycle”, “Wnt signaling” and “mesenchyme development” (Fig. 4D). Thus, we comprehensively identified the set of genes, the cardio-JAGs, as the building blocks for the juvenile specific functions of the young cardiomyocytes, as the new aspects to delineate the functionalities of the juvenile cells.

### Maturation dynamics in the cardiomyocytes

**Figure 5:**
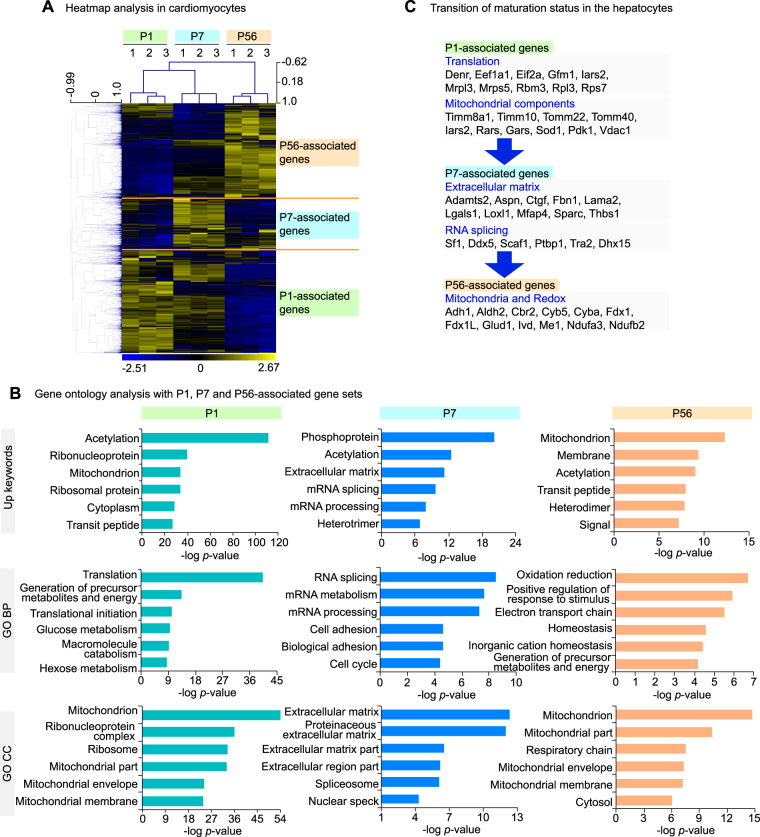
Maturation dynamics in the cardiomyocytes. (**A**) Heatmap analysis of transcriptome analysis in the cardiomyocytes at different ages. The distinct sets of genes were present as noted as P1-, P7- and P56-associated genes. (**B**) The GO analysis describing the transition of cellular functions in the cardiomyocytes. (**C**) A schematic for the transition of the cellular functions in the postnatal cardiomyocytes.

During the maturation phase in the heart, cardiomyocytes increase their number, hypertrophy and strengthen getting along with the increasing cardiac load as a result of the body size increment. To identify the genes responsible for the maturation, we focused on the gene sets distinctly expressed at each of P1, P7 and P56 (Fig. 5A). The comprehensive GO analysis revealed that the P1-associated genes were linked with translation-related functions such as “ribonucleoprotein” and “translation initiation”. The cellular components of mitochondrion were also enriched in P1. The P7-associated genes were linked with RNA processing-related terms such as RNA splicing and mRNA metabolism, and ECM-related terms such as “extracellular matrix” and “proteinaceous extracellular matrix”. The P56-associated genes were linked with mitochondrial functions such as “mitochondrion” and “electron transport chain” (Fig. 5B). These analyses reveal the dynamic transition in the cellular functions along the maturation of postnatal cardiomyocytes (Fig. 5C). Thus, the comprehensive gene expression profiling reveals the functional maturation dynamics and the pivotal genes characterizing each maturation stages in the postnatal cardiomyocytes.

### Identification of the common JAGs and their functions

**Figure 6:**
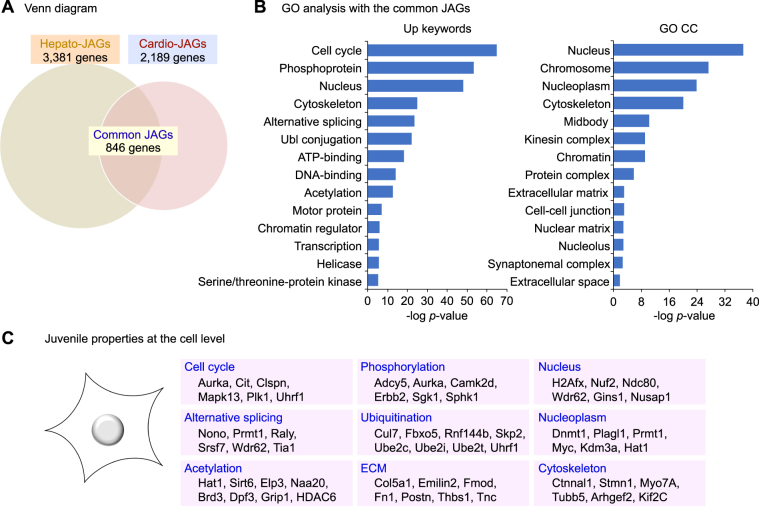
The common JAGs exhibit a distinct gene expression profile. (**A**) The Venn diagram showing the common JAGs as overlapping genes between the hepato-JAGs and the cardio-JAGs. (**B**) The GO analysis with the common JAGs. (**C**) The cellular functions that constitute the juvenile properties at the cell level.

To determine the genes universally relevant to establish the juvenile properties, we set our focus on the common genes overlapping in the hepato- and the cardio-JAGs (hereafter common JAGs). Consequently, we identified 846 genes as the common JAGs (Fig. 6A). We next performed the GO analysis to ask their functional enrichments. In addition to the expected terms such as “cell cycle”, “nucleus”, and “chromosome”, unexpected terms “alternative splicing”, “ubiquitination”, “acetylation” and “extracellular matrix” were significantly enriched in the common JAGs (Fig. 6B). This analysis clarified yet-investigated roles for the alternative splicing, the biochemical reactions such as phosphorylation, ubiquitination and acetylation, and the ECM in establishing the juvenile properties at the cell level (Fig. 6C). Thus, these analyses identified the common JAGs that underlie the juvenile properties of the young animals.

### JAGs associate with childhood-onset diseases

**Figure 7:**
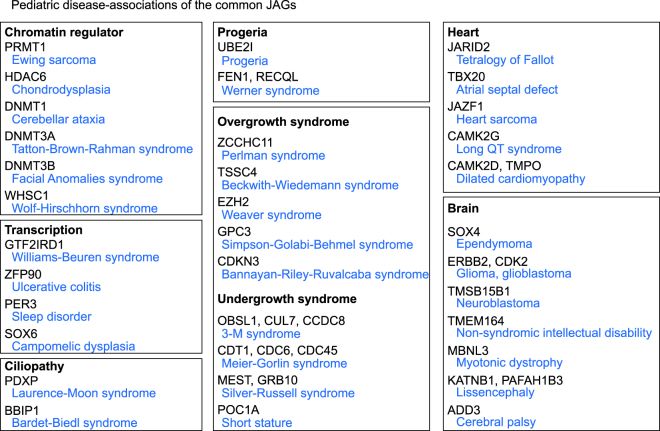
Associations of the common JAGs to the human genetic diseases. Biological categories of the genes and their associated human genetic diseases. All the common JAGs were searched for their association with human genetic diseases.

Our analysis identified the common JAGs that are predominantly expressed both in juvenile hepatocytes and cardiomyocytes. Based on the assumption if the common JAGs are essential for the juvenile properties, they should have strong connections to the pathogenesis of childhood-onset diseases. For that purpose, we tested the associations of the mutation in the common JAGs with pediatric diseases by testing whether a human genetic disease is reported as a result of a mutation of the particular gene. The rate for the successful annotation with a disease were higher in the common JAGs than all genes, indicating the common JAGs enriched the physiologically indispensable genes (Supplementary Figure S3). Notably, numerous growth-associated genes whose mutations cause overgrowth or undergrowth syndrome were included in the common JAGs (Fig. 7). In addition, genes that cause progeria were also included, specifically *UBE2I* for Progeria and *FEN1* and *RECQL* for Werner syndrome (Fig. 7), suggesting a role for the common JAGs to hamper the premature aging. Also, chromatin modifiers such as DNA methylases (*DNMT1*, *DNMT3A*, and *DNMT3B*), histone acetylase (*HDAC6*), histone methylase (*PRMT1*), a reader of histone modification (*WHSC1*), and transcription factors (*GTF2IRD1*, *ZFP90*, *PER3*, and *SOX6)* were included, suggesting a crucial role for chromatin regulation in maintaining the juvenile properties. The common JAGs responsible for heart diseases included: *JARID2* and *TBX20*, associated with a structural heart anomaly; *JAZF1*, associated with cardiac tumors; *CAMK2G*, associated with conduction defects; and *CMAK2D* and *TMPO*, associated with dilated cardiomyopathy. Thus, the common JAGs had dense connections to the childhood-onset diseases, including growth disorders and progeria syndromes.

We here identified the JAGs as the gene sets predominantly expressed in the juvenile hepatocytes and cardiomyocytes. Our analysis clarified their unique connections with biological functions such as alternative splicing, phosphorylation and ECM. These genes establish the juvenile properties of individual animals at the cell level, and consists important therapeutic targets as evidenced by the dense connection with the human genetic diseases.

## Discussion

Young animals exhibit distinct physiological properties that adults do not. But the molecular insights into the distinctiveness have not been completely elucidated. We here identified the JAGs as the molecular constituents of the juvenile properties through the comprehensive transcriptome analysis by high-throughput sequencing and their dense connections to the pathogenesis of childhood-onset diseases.

Growth is a hallmark of the juvenile properties. It is not completely understood how young individuals can increase their body size while adults cannot, and how the growth is stopped at the determined size. The cell division in young organs is unique in that it happens in well-differentiated functional cells, such as cardiomyocytes^18,19^ and pancreatic beta cells^50^. It is not fully understood how the differentiated cell duplicates and redistributes the highly-specialized chromatins and organelles. Uhrf1 recruits Dnmt1 maintenance DNA methylase to hemi-methylated genome DNA, thereby enabling the specialized chromatin modifications to be inherited^51,52^. Both Uhrf1 and Dnmt1 are indispensable for the maintenance of differentiated somatic cells^53–56^. Of note, both of *Uhrf1* and *Dnmt1* are the common JAGs. An identification of Uhrf1-interacting factors may uncover molecules engaged in the maintenance of differentiation status during the division of differentiated cells. This harbors particular importance considering regenerative medicine. Any compound that induces or enhances the proliferative activity of differentiated cells may establish a new regenerative medical approach as growth induction therapy.

Maturation is a postnatal functional refinement in organs to meet the demands increasing due to drastic changes at the birth and the body size increment later on. These changes include transitions in circulation; respiration; alimentation, in which nutritional paths switch from transplacental to oral; and body waste processing, which transitions from transplacental to hepatic. Maturation of an organ affects that of another organ^57^. It is not well understood how the juvenile cells adapt and self-transform themselves accordingly getting along with the changing necessities. Our transcriptome analysis revealed the functional dynamics reflecting the maturation in the juvenile cells. The GO analysis revealed the prominent gene sets that reflect the transition along the maturation stages. In hepatocytes, translation was followed by the expression of ECM genes and then redox genes, and in cardiomyocytes, translation was followed by the expression of ECM genes and then mitochondria genes. Further analysis might illuminate an involvement of ECM and a possible mechanism in controlling the organ size.

Numerous childhood diseases are caused by genomic mutations and therefore difficult to permanently treat. Our analysis based on transcriptome analysis identified numerous genes that consist the essential functional network in a cell, thereby constituting therapeutic targets. We assessed the physiological relevance of the common JAGs by investigating their associations to human genetic diseases. The common JAGs were more highly associated with the genetic diseases than unselected genes. This demonstrates the common JAGs are indispensable to maintain the juvenile homeostasis in a young cell. Three common JAGs were associated with progeria syndromes (*Ube2i*, *Fen1* and *Recql*). The association of the common JAGs with progeria syndrome implies the JAGs’ function to maintain the juvenescence. Also, the growth disorders had dense connections to the mutations of the common JAGs. An identification of mechanisms how the growth-associated JAGs regulate growth might give rise to a better understanding for the size control machinery, and a therapeutic idea for the growth disorders.

How do the JAGs exert their functions in a cell to achieve juvenescence? Stem cell or tissue progenitor cell compartments contribute to the establishment and homeostasis of the tissue architecture. The JAGs may possess functions in the tissue stem cells, especially in the organs in which the tissue stem cells are evidently identified, such as biliary systems^58^, brain^59^, hematopoietic cells^60^, skeletal muscles^61^, and others. Further elucidation of the machinery how the JAGs affect cellular functions may illuminate a role of the stem cells.

Genes associated with the RNA processing were also enriched in the JAGs. A gene is spliced to produce a structurally and functionally distinct protein in a context-dependent manner^62^. Further analysis as to the RNA splicing or RNA metabolism might lead to a new insight how the juvenile properties are established by these genes.

Our analysis illuminated a role of ECM in the juvenile properties. *Glypican3* is known to be indispensable for growth^63^ and a mutation in *Glypican3* causes Simpson-Golabi-Behmel overgrowth syndrome^64^. These findings clearly indicate ECM has an essential role in the organ growth, but we still do not know how the organ growth and the final size are strictly regulated by the ECM. Our analysis showed that numerous ECM genes are globally controlled during postnatal development. The further analysis utilizing cell, organoid, and animal models in which a select JAG is overexpressed or suppressed will facilitate our understanding toward how the organ size and the growth patterns are determined.

Numerous chromatin regulators were also included in the JAGs. As in the histone bivalency that is observed in undifferentiated pluripotent cells, a specific pattern of chromatin modifications characterizes developmental stages. The chromatin dynamics is considered to be more active in juvenile because of the larger requirements to differentiate and adapt to the external conditions, comparing to that in adult. These features of the dynamic chromatin state might underlie the functional plasticity of the juvenile organs, but further research is necessary to address this possibility.

P56-associated genes correspond to the genes repressed in the juvenile phase. In other words, the functions associated with the P56-associated genes, redox or mitochondrion, might have a suppressive role in the juvenile properties such as growth or developmental process. Further analyses from this viewpoint may further illuminate an unexpected role of the JAGs in the regulation of the juvenescence.

We hereby present juvenility-associated genes, JAGs, that establish the distinct properties of the juvenile animals. The JAGs possess functions such as alternative splicing, phosphorylation and ECM, setting these functions as the important bricks that constitute the juvenile properties of the young cell. The JAGs have dense connections with childhood-onset genetic diseases, indicating their physiological relevance and their potential as the new therapeutic targets.

## Materials and Methods

### Mice

All animal experiments were approved by the institutional animal care and use committee at Tokyo medical and dental university. All experiments were performed in accordance with the relevant guidelines and regulations. C57BL/6 N male mice aged at postnatal day 1 (P1), 7 (P7) and 56 (P56) were used. Hepatocytes and cardiomyocytes were isolated from the mice (n = 3) as described below.

### Isolation of Hepatocytes

Hepatocytes were isolated from P1 and P7 mice. Dissected livers were washed with phosphate buffer saline (PBS) briefly and treated with collagenase I (0.5 mg/ml) at 37 C for 30 min. After gentle resuspension, cells were pelleted by centrifuge at 100 g. The pelleted cells were resuspended and incubated with red blood cell (RBC) lysis buffer (155 mM NH4Cl, 12 mM NaHCO3, 0.1 mM EDTA) for 10 min, filtered through a 100 µm cell strainer and centrifuged at 100 g at room temperature (RT). Isolated cells were directly utilized for RNA extraction without putting the cells in culture, to avoid alterations introduced to cell status by culture. A part of isolated cells was utilized to determine the purity as consistently higher than 95%. The hepatocytes were isolated from P56 mice by the two-step liver perfusion method. Mice were anesthetized and portal vein was punctured. Inferior vena cava was cut through to allow for efficient perfusion. The liver was washed with the calcium- and magnesium-free Hank’s Balanced Salt Solution (CMF-HBSS) preheated to 37 C. The liver was digested via perfusion with pre-warmed digestive enzymes (collagenase, elastase and DNase)-containing buffer for 20 min. The liver was transferred into a culture dish filled with CMF-HBSS. Cells were dispersed and cell suspension was collected, filtered through a 100 µm cell strainer and centrifuged at 50 g for 1 min at RT. Isolated cells were directly utilized for RNA extraction without putting the cells in culture. A part of isolated cells was utilized to determine the purity and the viability by trypan blue exclusion as 95% and 92%, respectively. Images of cells were taken with EVOS FL cell imaging system (ThermoFisher Scientific).

### Isolation of Cardiomyocytes

Cardiomyocytes were isolated from the mice at P1 and P7 using the Primary Cardiomyocyte Isolation Kit (ThermoFisher Scientific). The purity of the cardiomyocytes at P1 and P7 was determined by the morphology and contraction as more than 90%. Isolated cells were not kept in culture and directly utilized for RNA extraction without putting the cells in culture. Images of a part of isolated cells that were put in culture were taken with EVOS FL cell imaging system (ThermoFisher Scientific). Isolation of the cardiomyocytes from the mice at P56 was performed as described previously^65^. The purity of the isolated cardiomyocytes at P56 was determined by the typical rod shape and contraction as more than 80%. Isolated cells were not kept in culture and directly utilized for RNA extraction without putting the cells in culture. Images of cells were taken with the EVOS FL cell imaging system.

### RNA-seq

A total RNA was extracted with TRIzol (Invitrogen). For the quality check of the extracted RNA, the RNA integrity numbers (RIN) were determined using Bioanalyzer (Agilent). cDNA library was prepared using TruSeq RNA kit (Illumina). The quality of the library was checked with Bioanalyzer and qPCR. The sequencing was done with HiSeq. 2000 (read length: 50 base pairs, single end; Illumina) to generate the Fastq files.

### Bioinformatics Analyses

The RNA-seq data was processed and analyzed using Galaxy (https://usegalaxy.org/)^66^. To determine the juvenility-associated genes (JAGs), the whole transcripts were filtered according to the expression levels higher than 0.8 (fragments per kilobase of transcript per million fragments sequenced, FPKM) and the fold changes higher than 2.0 both at P1 and P7 comparing to P56. The heatmaps were generated using MeV (http://mev.tm4.org). The expression was visualized using Integrative Genomics Viewer (http://software.broadinstitute.org/software/igv/). The gene ontology analysis was performed using the Database for Annotation, Visualization and Integrated Discovery (DAVID) v6.7 (https://david-d.ncifcrf.gov/). The gene set enrichment analysis (GSEA) was performed using the GSEA software^67,68^. The STRING network analysis was performed using the STRING database (http://string-db.org/)^69^. The annotations of the genes were obtained from GeneCards (www.genecards.org)^66,70^.

### Gene expression Analysis

Extracted RNA was quantified with NanoDrop Lite Spectrophotometer (Thermo Fisher Scientific) and reverse transcribed with High-Capacity RNA-to-cDNA kit (Applied Biosystems) and PCR Thermal Cycler Dice (Takara). Using the reverse transcribed cDNA as the template, qPCR was performed with LightCycler 480 SYBR Green I Master kit and LightCycler 480 instrument (Roche). The quality of specific amplification was assessed by the melting curve analysis. The data were normalized by mouse *Polr2a*. Primers used for qPCR are listed in Supplementary Table S1.

### Statistical analysis

For all quantified data, mean ± standard error of the mean (SEM) is presented. Statistical significance between two experimental groups is indicated by an asterisk and comparisons were made using the Student’s t-test. P-values less than 0.05 were considered significant.

### Data availability

The datasets generated during and/or analyzed during the current study are available from the corresponding author on reasonable request.

## Electronic supplementary material

Supplementary Information
