# Supplementary material for: Identification of juvenility-associated genes in the mouse hepatocytes and cardiomyocytes
Source: Sci Rep. 2018 Feb 15;8:3132. doi: 10.1038/s41598-018-21445-3 (PMC5814429; doi:10.1038/s41598-018-21445-3)

Supplementary Information

Identification of juvenility-associated genes in the mouse hepatocytes and cardiomyocytes.

Authors

[*]Faidruz Azura Jam[#], [*]Yosuke Kadota[#], [*]Anarmaa Mendsaikhan[#], [*]Ikuo Tooyama, [*,†]Masaki Mori

Affiliations

[*]Molecular Neuroscience Research Center (MNRC), Shiga University of Medical Science, Tsukinowa-cho, Seta, Otsu, Shiga, 520-2192, Japan.

[†]Department of Systems BioMedicine, Tokyo Medical and Dental University, 1-5-45, Yushima, Bunkyo-ku, Tokyo, 113-8510, Japan.

[#]These authors contributed equally to this work.

Supplementary Figures

Supplementary Figure S1. Appearance of the isolated hepatocytes and cardiomyocytes.

(A) The image of hepatocytes isolated from the P1 mouse taken one day after the isolation. Scale bar, 50 μm.

(B) The image of hepatocytes isolated from the P7 mouse taken one day after the isolation. Scale bar, 50 μm.

(C) The image of hepatocytes isolated from the P56 mouse taken one day after the isolation. Scale bar, 50 μm.

(D) The image of cardiomyocytes isolated from the P1 mouse taken one day after the isolation. Scale bar, 100 μm.

(E) The image of cardiomyocytes isolated from the P7 mouse taken one day after the isolation. Scale bar, 100 μm.

(F) The image of cardiomyocytes isolated from the P56 mouse taken on the day of isolation. Scale bar, 100 μm.

Supplementary Figure S2. Validation of RNA-seq results with quantitative PCR analyses.

(A) The quantitative PCR (qPCR) analysis of *Igfbp2*, a hepato-JAG, in the hepatocytes and cardiomyocytes. Data were normalized by *Polr2a*.

(B) The qPCR analysis of *Pleiotrophin*, a cardio-JAG, in the hepatocytes and cardiomyocytes. Data were normalized by *Polr2a*.

(C) The qPCR analysis of common JAGs in the hepatocytes and cardiomyocytes.

Data were normalized by *Polr2a*.

$*p < 0.05$, $**p < 0.01$, Student's t test. Data are represented as mean ± SEM.

Supplementary Figure S3. Association of the common JAGs to the human diseases.

The rates for the successful annotation of the common JAGs and all the genes to any human disease.

Supplementary Table S1. Sequences of the qPCR primers used in this study.

Gene	Forward	Reverse
Polr2a	5'-GAGTCCAGAACGAGTGCATGA-3'	5'-ACAGGCAACACTGTGACAATC-3'
Igfbp2	5'-CAGACCTCGGGTGAGAAAAG-3'	5'-CTGCTACCACCTCCCAACAT-3'
Pleiotrophin	5'-TTTTCATCTTGGCAGCTGTG-3'	5'-ACACTCCACTGCCATTCTCC-3'
Ezh2	5'-CCTGTTCCCACTGAGGATGT-3'	5'-GAGCCGTCCTTTTTCAGTTG-3'
Gpc3	5'-ACGGGATGGTGAAAGTGAAG-3'	5'-AGGTGGTGATCTCGTTGTCC-3'
Uhrf1	5'-ACGGTGCCTACTCATTGGTC-3'	5'-GCTTCTGGTCAGAGGACTGG-3'
Postn	5'-TGGTCACTTCACGCTCTTTG-3'	5'-GCCACTTTGTCTCCCATGAT-3'
Map4k4	5'-CTGGGTCCATCACAGACCTT-3'	5'-TCGGTGAATAACGTGGTGAA-3'
Prmt1	5'-GCCTGCAAGTGAAGAGGAAC-3'	5'-CTCAGGACTGGTGGAGAAGC-3'
Tia1	5'-AGATGCCCGTGTGGTAAAAG-3'	5'-TTCTGCATCCCATTTGTTGA-3'
Sirt6	5'-CCTGTAGAGGGGAGCTGAGA-3'	5'-GAGGTACCCAGGGTGACAGA-3'

Supplementary Figure S1. Appearance of the isolated hepatocytes and cardiomyocytes.

Images of the isolated hepatocytes

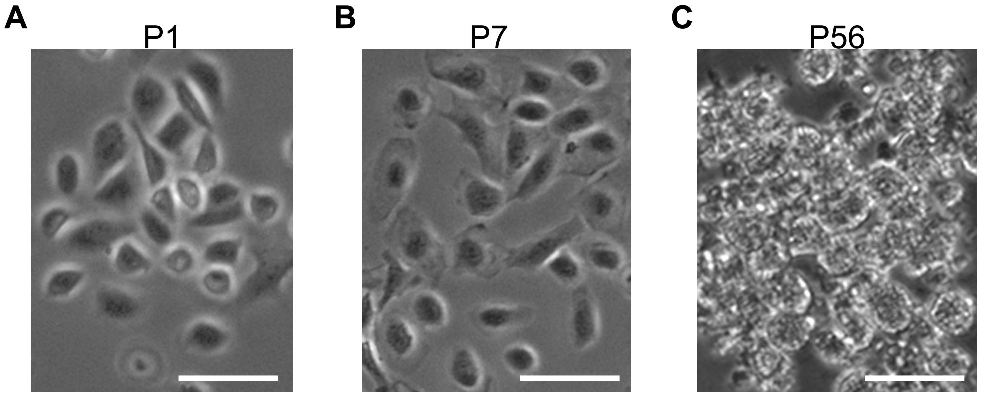

Images of the isolated cardiomyocytes

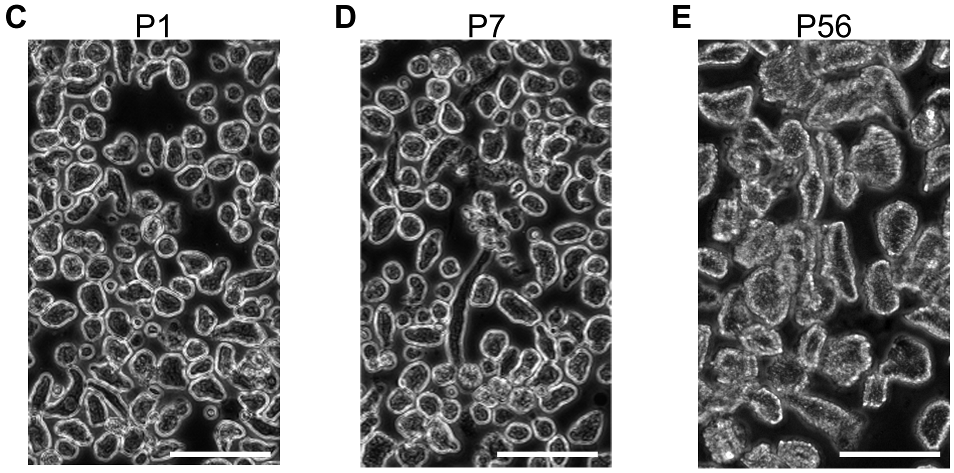

Supplementary Figure S2. Validation of RNA-seq results with quantitative PCR analyses.

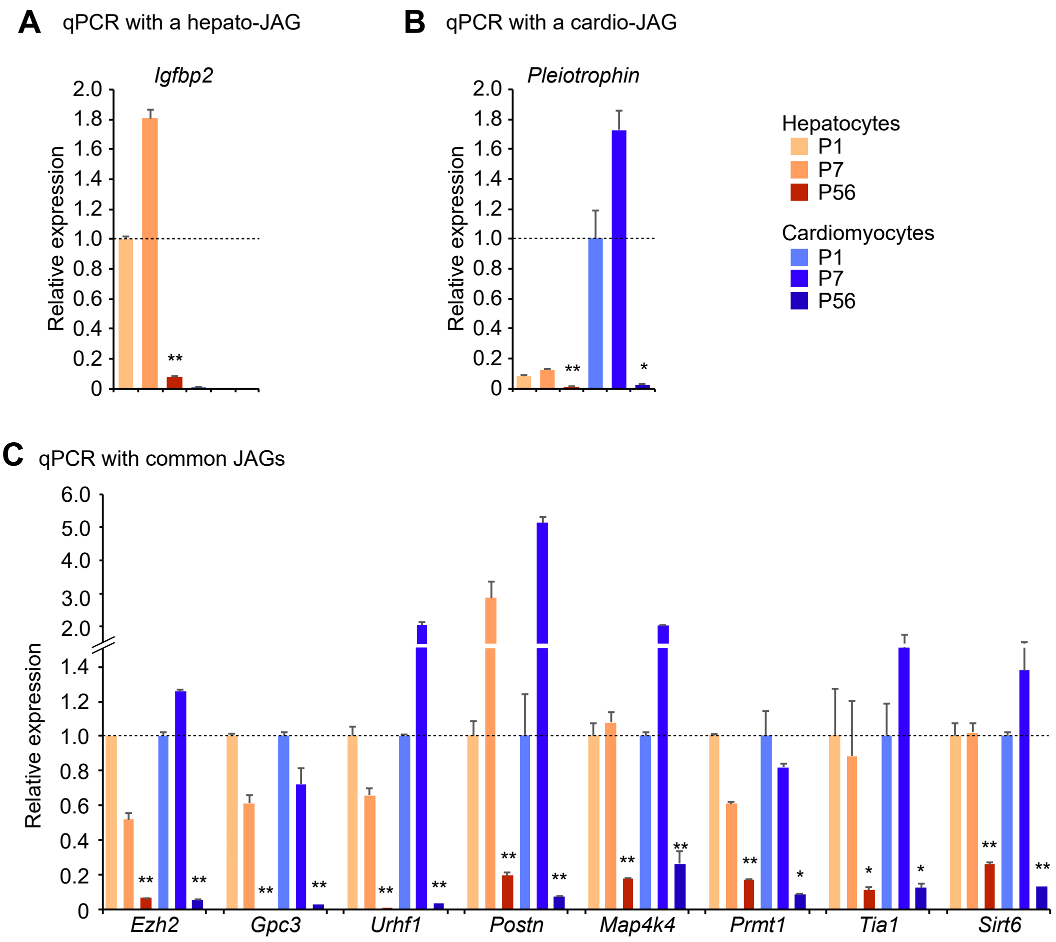

Supplementary Figure S3. Association of common JAGs with human diseases.

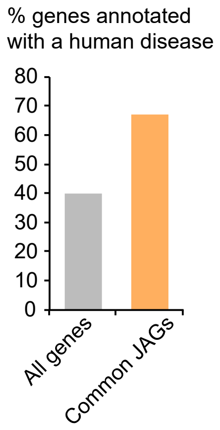

Supplement: Supplementary file 1 — Supplementary Information [file 41598_2018_21445_MOESM1_ESM.pdf]
